# Evaluation of cuspal deflection and microleakage of an incrementally packed versus a low shrinkage bulk-fill nanohybrid resin composite: an in-vitro study

**DOI:** 10.1186/s12903-026-09190-w

**Published:** 2026-07-15

**Authors:** Rahma Fares Shaban, Rabab Ismail Abdel Fattah, Rehab Khalil Safy

**Affiliations:** Department of Conservative Dentistry, Faculty of Dentistry, Suez Canal University, Ismailia, Egypt

**Keywords:** Dental restoration, Resin Composites, In Vitro Techniques, Cuspal deflection, Bulk-fill composite, Microleakage

## Abstract

**Background:**

Despite the numerous advantages of using resin-based composite restorations, polymerization shrinkage and the resulting stresses remain significant concerns. These stresses are associated with two critical clinical outcomes: cuspal deflection and microleakage. This study aimed to evaluate the cuspal deflection and microleakage of an incrementally packed nanohybrid and a low-shrinkage bulk-fill resin composite.

**Methods:**

To assess cuspal deflection, 40 maxillary premolars were randomly divided into two groups (*n* = 20). Standardized mesio-occluso-distal (MOD) cavities were prepared, and one group was restored with incremental Filtek Z250XT and the other with the low-shrinkage bulk-fill resin composite Grandio^®^SO x-tra. Cuspal deflection was measured after cavity preparation and five minutes post-restoration using a universal horizontal metroscope. For evaluation of microleakage, each group was further subdivided into non-thermocycled and thermocycled subgroups. Microleakage was assessed using a 2% methylene blue dye solution at 37 °C for 24 h, followed by sectioning and examination under magnification.

**Results:**

The highest mean cuspal deflection was recorded in the incremental group (12.80 ± 2.57 μm) compared to the bulk-fill group (9.60 ± 2.41 μm). A statistically significant difference in cuspal deflection was observed between the two groups (*p* = 0.010). No significant difference was found in microleakage scores between the incrementally packed and bulk-fill groups (*p* = 0.305). The incrementally packed group exhibited the highest mean microleakage score (259.66 ± 20.53 μm), while the bulk-fill group had the lowest mean score (251.10 ± 15.36 μm). Regarding the effect of thermocycling on microleakage, regardless of the type of resin composite used, a statistically significant difference was noted between the non-thermocycled subgroup (241.12 ± 10.17 μm) and the thermocycled subgroup (269.63 ± 11.97 μm) (*p* < 0.001).

**Conclusion:**

Low shrinkage bulk-fill composite reduced cuspal deflection in comparison to the incrementally packed materials. Bulk-fill and incrementally packed resin composites both showed comparable vulnerability to microleakage. The thermocycling procedure exerted a deteriorating effect on marginal integrity.

## Background

Increased demand for posterior tooth-colored restorations has driven advances in resin-based composites (RBCs), improving both aesthetics and clinical performance [1]. However, polymerization shrinkage and resultant stresses remain critical concerns [2], primarily causing cuspal deflection and microleakage [3]. When bond strength is sufficient to withstand these stresses, cusps are pulled inward, reducing intercuspal distance and generating internal stress without detachment [4]. Cuspal deflection can cause enamel cracks, postoperative sensitivity, crazing, microcrack propagation, decreased fracture resistance and a consequent cusp fracture in extreme cases [5]. This deflection is influenced by cavity dimensions, wall thickness, and the RBC’s volumetric contraction and modulus of elasticity [6]. Conversely, when shrinkage stresses exceed bond strength, the composite-tooth interface degrades, creating marginal gaps that promote staining and secondary caries [7]. These complications are most pronounced in large mesio-occlusal-distal (MOD) cavities due to reduced tooth rigidity and high C-factors [3, 8].

To mitigate these stresses, traditional incremental techniques limit RBC layers to 2 mm [9]. Incremental placement improves depth of cure while reducing polymerization shrinkage stress. However, this procedure can be time consuming and highly technique sensitive [10].

While new bulk-fill RBCs address the time-sensitivity of incremental packing by allowing 4 mm increments [11], the increased depth of cure was achieved by introducing novel photoinitiators, adjusting filler-matrix interface, associated with filler reduction, enlarged filler size, and addition of stress relieving monomers [9, 11, 12].

It remains unclear whether the “stress-relieving” chemistry of these materials can truly overcome the high C-factor demands of large MOD cavities. Although the manufacturer reports minimal shrinkage (1.4%) for nanohybrid bulk-fill RBCs Grandio^®^SO x-tra, independent comparative data on their performance in high-stress, large-volume restorations is limited. While previous studies have evaluated other commercial bulk-fill RBCs, investiagtions focusing on both the microleakage and cuspal deflection of this low-shrinkage nanohybrid RBC in comparision to incremental RBCs remain limited. Therefore, the current study aimed to evaluate the cuspal deflection and microleakage of Grandio^®^SO x-tra compared with an incrementally placed nanohybrid RBC in standardized MOD preparations.

The first null hypothesis of the current study was that there is no significant difference between incrementally packed and bulk-fill RBCs regarding cuspal deflection. The second null hypothesis was that there is no significant difference in microleakage values between the two types of RBCs.

## Materials and methods

### I-Materials

The materials’ brand names, descriptions, compositions, manufacturers, and batch numbers are presented in Table 1.

**Table 1:** Materials used in the study

Material brand name	Description	Composition	Manufacturer	Batch numbers
All-Bond Universal^®^	Universal Adhesive	10-MDP di-methacrylate resin, HEMA, ethanol, water, Initiators	Bisco, Shaumburg, USA	220,007,024
Filtek™ Z250 XT	Nanohybrid RBC Shade A2	Matrix: Bis- GMA, UDMA, Bis-EMA, TEGDMA Filler: Inorganic fillers: zirconia, silica 81.8% (wt%), 60% (vol%)	3 M ESPE St.Paul, M, USA	NE58799
Grandio^®^ SO x-tra	Bulk-fill nanohybrid RBC Shade A2	Matrix: Bis-GMA, Bis-EMA, TCDDMA Filler: Inorganic filler, organically modified silica.86% (wt%)	Voco, Cuxhaven, Germany	2,306,551

### II- Methods

#### II-1 Study design and sample size calculation

Following ethical waiver issued by the Research Ethics Committee of the Faculty of Dentistry, Suez Canal University (No. 602/2023), this in vitro study was performed. A minimum total sample size of 40 specimens was required to detect an effect size of 0.81 with 80% power (1 − β = 0.80) at a significance level of *p* < 0.05 and a partial eta squared of 0.13. Sample size was calculated using G*Power software (version 3.1.9.6) [13–15]. An effect size of 0.81 is considered large according to standard Cohen’s d benchmark, indicating more pronounced differences between groups. A partial eta squared of 0.13 is considered a medium-to-large effect, indicating a substantial impact on the results. According to the sample size calculation, each restoration group (M1 and M2) included 20 specimens, and each subgroup (e.g., M1T0 and M1T1) included 10 specimens (*n* = 10) [16]. The interaction between them was detected using two-way ANOVA.

The effect size was calculated according to the formula:

$$\begin{array}{c}d=\frac{\sigma_\mu}\sigma\\\sigma_\mu^2=\frac{{\displaystyle\sum_{i=1}^k}\;n_j\;\left(\mu_i-\mu\right)^2}N\end{array}$$

#### II-2 Selection of teeth

In this study, according to the sample size calculation, 40 human maxillary premolars extracted for orthodontic reasons were utilized. Patients were chosen within the age range of 18–36 years based on eligibility criteria [17]. Selection of teeth was carried out according to the inclusion criteria, where selected teeth were visually examined to be caries-free with no visible cracks or abrasions on all external surfaces and unrestored previously [18].

Following extraction, teeth were rinsed under copious running water to debride blood and mucus, then they were scaled to remove calculus and any attached periodontal ligament tissue. Finally, teeth were polished with fine pumice and soft rubber cups mounted on a slow-speed handpiece powered by a micromotor (Strong 204, Saeshin, Korea) at a speed of 3000 rpm [19].

The selected teeth had regular and almost similar occlusal anatomy along with standard crown dimensions. Standardisation of dimensions was performed using a digital caliper (Mitutoyo, Kawasaki, Japan) to check the measurements of each tooth. Teeth from patients outside the target age range or with dimensions outside the predefined ranges were excluded [20]. The teeth were then stored in normal saline containing 0.5% thymol as an antifungal agent and used within three months [21].

#### II-3 Teeth mounting and preparation

In order to simulate the biological width and replicate alveolar bone support of sound teeth, root surfaces were marked 2 mm apical to the cementoenamel junction [22–24]. Auto-polymerizing acrylic resin (Acrostone, Egypt) surrounded by a custom-made silicone mold (internal diameter 16.5 mm, external diameter 21.5 mm, and height 9.6 mm) was used to embed the teeth in it, maintaining their long axis parallel to the long axis of the silicone mold [25].

Teeth were randomized using a computer-generated randomization sequence generated by Random Allocation Software (Version 2.0). To minimize bias, allocation was concealed, and an independent colleague (not involved in the testing) assigned the teeth to their respective groups. Random numbers from (one to 40) were given for each tooth. The teeth were equally allocated into two groups (*n* = 20) according to the RBC material used for restoration. All teeth received standardized MOD cavities using a parallel-sided, flat-ended diamond fissure bur No. SF-41 (Mani, Inc, Japan). The bur was mounted on a high speed handpiece (Sirona, Germany) under continuous water cooling [20]. The cutting bur was replaced after every five preparations to avoid dullness and ensure efficient cutting. The same operator prepared all cavities to ensure standardization [26]. Cavity dimensions were evaluated with the aid of a digital caliper and periodontal probe and were standardized as follows: The bucco-palatal width of each cavity was 3 mm, and the depth was measured 4 mm from the cavo-surface margin to the pulpal floor. Moreover, buccal and lingual walls were kept nearly parallel to each other by using the same parallel-sided bur with a 90º cavosurface margin and rounded internal line angles of the cavity. Finally, depth and width of cavities were rechecked using digital caliper. Then, specimens were stored for 24 h in distilled water before cuspal deflection evaluation.

#### II-4 Assessment of cuspal deflection

After cavity preparation, cuspal deflection was evaluated by attaching two small glass beads to serve as cuspal indices. One index was fixed to each cusp as a reference point for intercuspal distance measurements [27]. Fixation of the cuspal indices was carried out within small shallow concavities that were prepared using high-speed round bur (BR-31, Mani, Inc., Japan). For each specimen, the concavities were prepared on the outer surface within the enamel at 1 mm below both cusp tips. These concavities allowed secure seating of glass beads ensuring stable and reproducible measurements. Then, these cuspal indices were fixed with the aid of α- Cyanoacrylate adhesive (Amir Alpha, Cyanoacrylate Adhesive, Cairo, Egypt). Consequently, the intercuspal distance of each specimen of both groups was measured using a universal horizontal metroscope [28, 29] (Octagon, Gold, Pune Maharashtra, India). During this procedure, the device measuring probe contacted the cuspal indices under constant measuring force and the intercuspal distance was measured and recorded for both groups after cavity preparation (C1) in micrometers (µm) [28]. Intercuspal distance measurements were repeated three times and the mean of the three values was calculated and used for statistical analysis. All measurements were made by a single operator in a temperature-controlled room.

After measuring the initial cuspal deflection, to circumscribe the prepared cavity of each tooth, a tofflemire metal matrix holder was securely adapted [30]. The top margin of the band was placed at 1 mm above the occlusal margin. The tofflemire matrix band was contoured and secured tightly, surrounding the tooth being restored by tightening the retainer to the point at which resistance was initially felt. To avoid overfilling at the gingival margins of the cavity, the matrix was tightened and held by finger pressure against the gingival margin [31].

Subsequently, the cavity was rinsed thoroughly with water and then dried gently with gentle air. According to the manufacturer’s instructions, an absorbent cotton pellet was used to maintain a moist dentin surface by blotting excess moisture from the cavity [32]. Two coats of universal adhesive (All Bond Universal^®^, Bisco Inc., USA) were applied with a saturated microbrush tip to the enamel and dentin and then scrubbed for 10–15 s per coat. Compressed air was directed gently for 10 s to allow penetration of the adhesive system and evaporation of the solvent until the surface appeared uniformly glossy. According to the manufacturer’s instructions, the adhesive was polymerized for 10 s using an LED light curing unit (Elipar S10, 3 M, ESPE, St Paul, MN, USA) set to default mode at light intensity 1200 mW/cm^2^ and wavelength 430–480 nm. The light curing tip was maintained as close as possible to the tooth surface to ensure complete polymerization [23].

Then, the incrementally packed group (M1) was restored using the nanohybrid RBCs (Filtek™ Z250 XT, 3 M ESPE, St.Paul, Minnesota, USA). The group was restored in successive 2 mm oblique increments restoring the entire cavity [33]. Each increment was adapted using a ball and pear- shaped instrument (Brasseler, USA) and then light cured from the occlusal aspect for 20 s following the manufacturer’s instructions. Meanwhile, the bulk-fill group (M2) was restored using a bulk-fill nanohybrid layer of RBCs (Grandio^®^SO x-tra., Voco, Cuxhaven, Germany). This RBC material was applied as a single 4 mm increment to fill the whole cavity, followed by curing for 20 s as instructed by the manufacturer.

Further curing for 40 s was done following the removal of the tofflemire band and retainer from the occlusal, buccal, and lingual aspects to achieve adequate polymerization of the composite resin of all specimens of both groups [34]. Five minutes after restoring the specimens, the intercuspal distance of both groups was re-evaluated (C2). Each measurement was repeated three times and the mean of the three values was recorded as (C2) for each sample. The incrementally packed group was recorded as (M1C2) and the bulk-fill group as (M2C2). Cuspal deflection (ΔC) was calculated as the difference between C1 and C2 using the following equation:

$\:\varDelta\:$C = Initial intercuspal distance – Intercuspal distance after five minutes from restoration.

#### II-5 Microleakage assessment at the gingival margin

After measuring the cuspal deflection values, all specimens were gently demounted from the resin blocks and then dried thoroughly. Then, each restored group was further subdivided for microleakage assessment into two subgroups: non-thermocycled (T0) or thermocycled (T1) [22, 35]. Thermocycled specimens underwent 5000 cycles between water baths of 5 °C and 55 °C with a dwell time of 20 s and a transfer time of 10 s [36]^,^ using a thermocycler (MPM Instruments, Bernareggio, MI, Italy).

Evaluation of microleakage was evaluated after covering the apices of all specimens with wax. Then, the whole surface was coated with nail varnish using two coats to prevent dye penetration through surface cracks from the external surface of the tooth, except for 1 mm around the margin of the restoration [37]. Subsequently, all specimens were submerged in 2% methylene blue dye for 24 h at room temperature, followed by rinsing thoroughly under running water and air-drying [38]. Immediately after washing, specimens were sectioned longitudinally in a mesio-distal direction in the middle of the restoration at low speed with copious water coolant. Sectioning was performed using a double-faced diamond disc mounted to a precision sectioning saw (Isomet 4000, Buehler Ltd., Lake Bluff, USA). The deepest dye penetration at the gingival margin for each specimen was evaluated at 40x magnification using a stereomicroscope (Nikon MA100 stereomicroscope, Japan). Each image of the restoration was captured and then transferred to a computer and analyzed with an image analysis software program (Image J, NIH Image, USA), and the leakage was measured in micrometers (µm) [19, 39, 40] by a blinded single examiner to reduce variability. Stored digital images were re-evaluated after two weeks to ensure reliability.

#### II-6 Statistical analysis

In order to assess the normal distribution of specimens, Shapiro-Wilk test was used to assess the data distribution. All experimental groups showed a normal distribution *p* > 0.05. Data are presented as mean ± standard deviation (SD). To compare the two groups (Bulk-fill and incrementally packed group) for cuspal deflection, independent-samples T-test was used. Two-way ANOVA was used to compare (Non-thermocycled and Thermocycled groups) and their interaction with the restorative material for microleakage results. Statistical significance was considered at p-value ≤ 0.05 using SPSS software for Windows version 26.0 (Statistical Package for the Social Sciences, Armonk, NY: IBM Corp).

## Results

### I-Cuspal deflection results

**Fig. 1:**
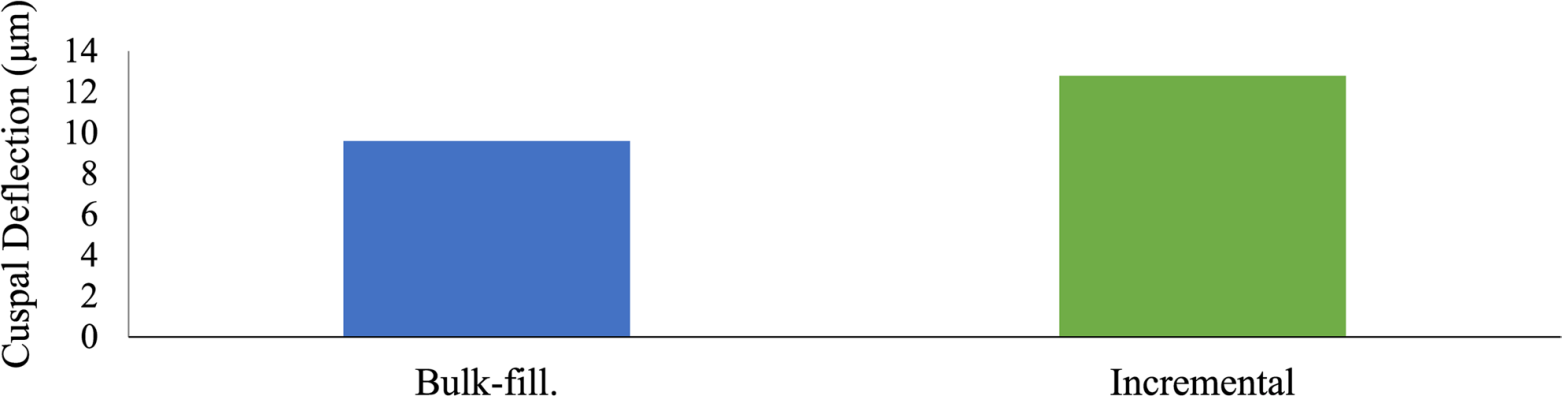
Cuspal deflection values for bulk-fill and incrementally packed RBCs

The results of the current study showed that both groups exhibited a degree of cuspal deflection, with a statistically significant difference between incrementally packed (M1) and bulk-fill RBCs (M2) using independent samples T-test (T = 2.86; *P* = 0.010). The highest mean value was recorded in the incrementally packed group (M1) (12.8 ± 2.57 μm) compared to the bulk-fill group (M2) (9.60 ± 2.41 μm), as shown in Table 2; Fig. 1.

**Table 2:** The mean, standard deviation and significance values of cuspal deflection of incremental and bulk-fill group

∆c	Incremental	Bulk-fill	Independent T-test	*P* value
Mean	12.80	9.6	2.86	0.010**
SD	2.57	2.41		

**; mean significant difference *p* < 0.05

### II-Microleakage results

#### II-1 Effect of type of RBCs on microleakage

Evaluating the effect of utilized RBC material on microleakage results regardless of thermocycling, results showed that there was no statistically significant difference found between the incrementally packed group (M1) and the bulk-fill group (M2). Generally, the highest mean value was recorded in the incrementally packed group (M1) (259.66 ± 20.53 μm) compared with the lowest mean value in the bulk-fill group (M2) (251.10 ± 15.36 μm), with a *p* = 0.305 indicating non-significance Table 3.

#### II-2 Effect of thermocycling on microleakage

**Fig. 2:**
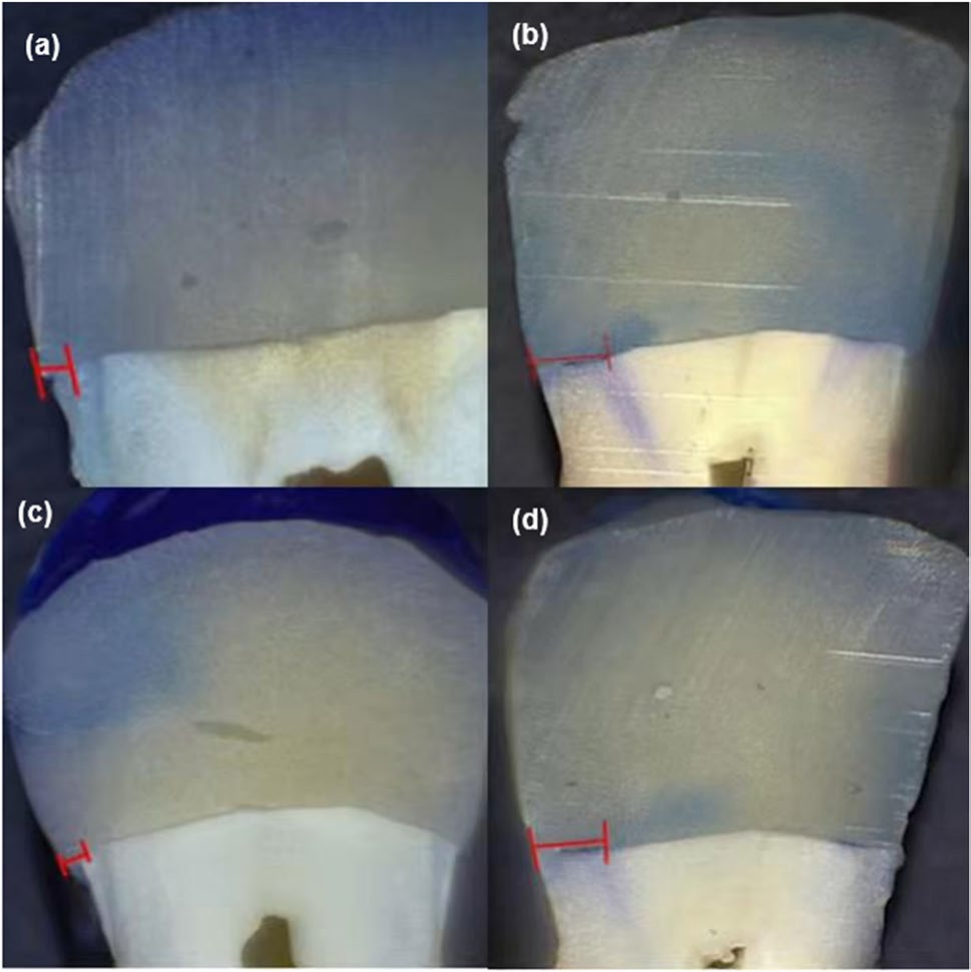
Depth of dye penetration at gingival margin. Arrows indicate extent of linear penetration along cervical margin at 40x magnification. **a**- non-thermocycled incrementally packed group. **b**- thermocycled incrementally packed group. **c**- non-thermocycled bulk-fill group. **d**- thermocycled bulk-fill group

**Fig. 3:**
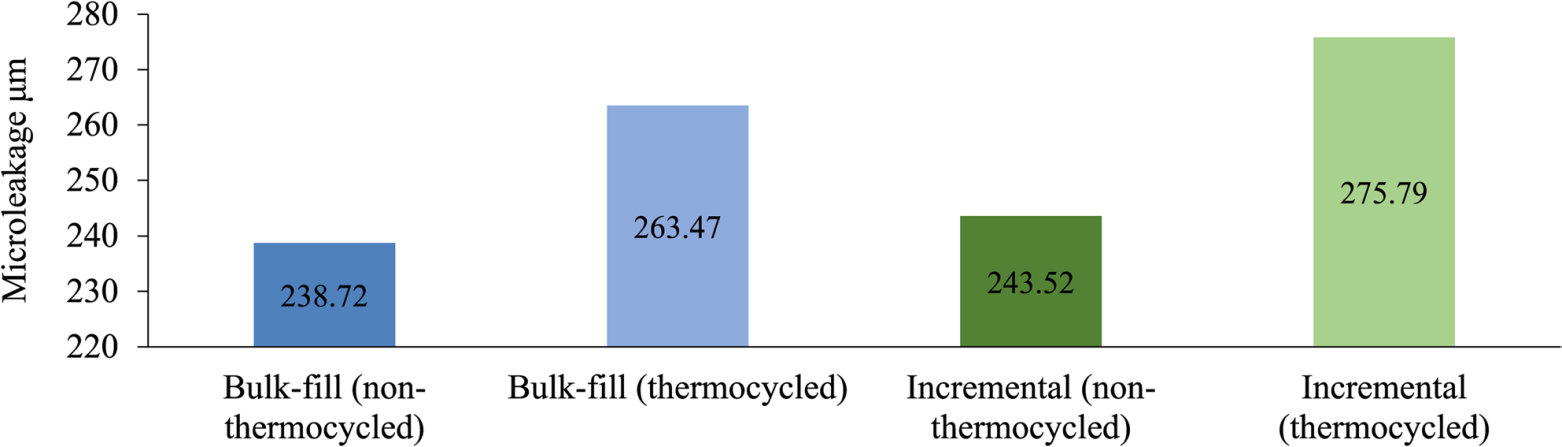
Bar chart representing microleakage values for all tested subgroups

Concerning the effect of thermocycling on gingival microleakage values regardless of the type of utilized RBC, the results of the present study revealed that a statistically significant difference was found between non-thermocycled (T0) and thermocycled (T1) subgroups at (*p* < 0.001). Generally, the higher mean values were recorded in the thermocycled subgroup (T1) (269.63 ± 11.97 μm) compared with the non-thermocycled subgroup (T0) (241.12 ± 10.17 μm), (Table 3; Figs. 2 and 3).

**Table 3:** The mean and standard deviation values for microleakage of all tested subgroups

	Thermocycling				
Material	Non-Thermocycled			Thermocycled	
	Mean	SD		Mean	SD
Bulk-fill	238.72^b^	9.64		263.47^a^	7.43
Incrementally packed	243.52^b^	11.20		275.79^a^	13.12
Two-way ANOVA P values					
Material (M)			0.305		
Thermocycling (T)			< 0.001**		
M x T			< 0.001**		

**; mean significant difference using two-way ANOVA at *P* < 0.05

The letter (a) indicates the highest average (highest subgroup)

The letter (b) gives the following average value

The similarity of letters between subgroups indicates that there are no significant differences between subgroups that had the same letter

The different letters between the subgroups mean that all the subgroups are significantly different from each other

## Discussion

Over the years, various strategies have been suggested to reduce polymerization shrinkage and its clinical consequences, such as cuspal deflection and microleakage [4]. One of the most prevalent complications that arise as a result of the polymerization stresses of resin-based composites on tooth structure is cuspal deflection. Cuspal deflection acts as a preloading that facilitates tooth fracture under tension [23]. This occurs particularly in MOD cavities due to decreased tooth stiffness after removal of marginal ridges [41]. Loss of enamel continuity during cavity preparation could consequently lead to tooth deformation, dentin deformation, and cuspal deflection [42].

Additionally, lack of adaptation at the tooth-restoration interface and the occurrence of marginal microleakage because of dimensional changes is a major concern. Microleakage, which is not clinically detectable, represents bacterial passage, liquids, molecules, and ions across the interface between the cavity wall and restoration through interfacial gaps [37]. Several restorative dentistry failures are a result of those interfacial gaps as they accelerate material deterioration, resulting in a shorter restorative life. Moreover, it can also result in permanent impairment of the integrity of hard tooth structure, marginal discoloration, secondary caries, postoperative pain, and pulpal involvement [43]. Microleakage surrounding restorations is apparently a multifaceted process rather than a single entity. The ionic charge, diffusing fluids’ chemical reactivity, the physical and chemical characteristics of restorations along with operator skills are critical factors in causing microleakage [38].

Various methods were proposed to reduce these consequences, such as liner placement and modifying the light curing technique. Also, oblique incremental application has been utilized to minimize polymerization shrinkage stresses, cuspal deflection, and its consequences as reported in literature [44]. However, it also has its drawbacks as it is time-consuming and technique-sensitive especially with an increased number of increments in large cavities [9].

On the other hand, bulk-fill RBCs were introduced to the market as one of the proposed efforts to decrease polymerization shrinkage stresses thereby reducing cuspal deflection. These materials are formulated to allow their application in 4 mm increments, thus minimizing voids between layers and decreasing the need for layering techniques, thereby saving time while still maintaining proper mechanical properties [45]. This improvement in the depth of cure is attributed to several factors, such as increased translucency and the incorporation of supplementary photoinitiator systems, as proved by various studies [10].

In the current study, to exclude the problem related to compliance of the testing system and supporting structures, human extracted teeth were selected [46]. Maxillary premolars were specifically chosen for this study due to their high similarity in shape and size. Moreover, the buccal and palatal cusps are nearly at the same level, nearly consistent in size, and centrally positioned relative to each other [20]. In addition, the anatomical crown form of maxillary premolars facilitates cusp fracture under occlusal loads, thus making the effect of cuspal deflection most apparent in them compared to other dentition. Crown volume and crown/root proportion of the maxillary premolars may also contribute to their high incidence of cuspal deflection [47].The selected teeth were standardized and teeth were measured and designated to treatment groups so that the mean bucco-palatal and mesio-distal width of all specimens varied within a slight range.

In this study, large MOD cavities were prepared in a standardized manner to receive RBC restorations, as removal of marginal ridges resulted in decreased tooth rigidity. Additionally, greater cuspal deflection occured in larger cavities as larger restorations generate heightened stress on the cuspal area [8]. Therefore, under circumstances that accentuate these factors, this choice of preparation was made to evaluate the cuspal deflection. Cavity depth was standardized to 4 mm in depth from the cavosurface margin to the pulpal floor, corresponding to the maximum depth of cure of bulk-fill RBCs. This was expected to increase cuspal deflection allowing more prominent simulation of clinical situations [48]. This could be attributed to the fact that removal of more tooth structure in extensive cavities causes more flexibility of the cusps and more compliance with RBC shrinkage. Moreover, the effect of polymerisation shrinkage stresses increases as a result of increased cumulative volume of the RBCs required for restoring larger cavities [8]. It was also reported that complete recovery of cusps to their original position occurred with small cavities. This recovery is mostly due to hygroscopic expansion in the oral cavity. On the contrary, complete recovery did not occur with large cavities [49].

Based on the literature, recording baseline measurements following cavity preparation may lead to higher cuspal deflection values because of pre-existing residual stresses within the tooth. It remains unclear whether these stresses are naturally present in teeth or result from extraction or storage procedures. That is why the baseline intercuspal distance was recorded after cavity preparation in this study [20].

Universal horizontal metroscope is considered an optical method capable of detecting micrometer-scale changes in intercuspal distance [28, 29], offering quantitative and comparable data, and thus was used in this study. However, the device measures only linear distance thus 3D deformation patterns cannot be captured. Minor changes in reference points can induce measurement errors, and operator-dependent alignment is a limitation of this method. Other accepted methods have been used to assess cuspal deflection, each with distinct advantages and limitations. Direct-contact displacement transducers offer high- resolution measurements but are sensitive to mechanical alignment and tooth angulation. Strain gauges record surface strain rather than cusp deflection, which may underestimate tooth flexural behavior. Conventional micrometers are simple and affordable but lack the micrometer-scale resolution needed to assess cuspal deflection reliably. More advanced digital image/laser-based scanning systems can produce map detailed deformation patterns, but are expensive, difficult to standardize, and technically complex.

Cuspal deflection was assessed after curing five minutes as multiple studies concluded that during this period maximum inward displacement occurred. This may be attributed to the continued polymerization process in the presence of residual free radicals in this period and the persistence of double bonds’ reaction within RBC restoration for a few minutes following cessation of light irradiation [27].

It was mentioned that bulk-fill RBC materials are potentially effective in achieving remarkably low polymerization shrinkage, clinical simplicity, enhanced depth of cure (≥ 4 mm) and improved physical properties [23]. Recently, a new bulk-fill RBC material with the manufacturer’s claim of having minimal polymerization shrinkage stresses was launched. Therefore, in this study, the new bulk-fill composite material (Grandio^®^SO x-tra) was used to be compared with the traditional incrementally packed RBCs [50].

The results of the current study revealed that cuspal deflection in unrestored MOD cavities showed no significant difference between both tested groups. Also, the results showed that both restored groups had a degree of cuspal deflection due to the occurrence of polymerization shrinkage, though with varying degrees. The results also showed that bulk-fill RBCs had significantly lower cuspal deflection than incrementally packed RBC material.

The difference in cuspal deflection values of the bulk-fill and the incrementally packed RBCs in the current study could be attributed to the different chemical composition of both tested materials. It was also reported that conventional RBCs had higher post-gel shrinkage than bulk-fill composites.

This result could be related to the bulk-fill RBC material used in the present study, characterised by minimal polymerization shrinkage (1.4%) whereas Filtek™ Z250 XT showed approximately (1.6%) polymerization shrinkage. It is worth noting that the shrinkage during RBCs polymerization is the main force inducing cusp deflection. Research indicated that the degree of polymerization shrinkage and cuspal deflection are highly linked. The materials that showed a higher shrinkage value also exhibited more cuspal deflection [51]. This difference in polymerization shrinkage could possibly explain the difference in the cuspal deflection value in both groups.

On the other side, there is an inverse correlation between the degree of volumetric shrinkage of RBCs and the percentage of inorganic filler particles incorporated [52]. Translucent bulk-fill materials usually achieve their translucency through optimized filler loading and refractive index matching between filler and resin [53]. Predominantly, higher filler load embedded in the resin matrix leads to decreased availability of monomers for the polymerization reaction, which results in decreased shrinkage of the RBCs [9]. Grandio^®^SO x-tra consists of a special composite formulation, which contains about (86% wt%) of fillers, which is higher compared to the filler content of Filtek™ Z250 XT (81.8% wt%). Therefore, the difference in the cuspal deflection among the two tested RBC materials used in the current study could be explained by the difference in the filler loading [9].

Relaxation of polymer chains occurs with the decrease in polymerizing light intensity, which in turn decreases with the decrease in the distance of the light-curing tip to the RBC. Hence, less contraction stresses are produced in the deepest portions of the cavity when bulk filled, permitting a delay in the attainment of the gel point. This delay leads to a prolonged pre-gel phase, allowing internal stresses generated by the shrinking polymer chains to be relieved by molecular flow, thus reducing polymerization stresses [54].

These results are consistent with Demirel et al., Lins et al., Politi et al., and Elsharkasi et al., [45, 55–57].

On the other hand, the results of this study were inconsistent with Tsujimoto et al., who reported similar cuspal deflection values with no significant difference between conventional and bulk-fill RBCs. This may be due to difference in methodology, where aluminium blocks were used instead of extracted teeth and evaluated cuspal deflection with the use of a micrometer and a Confocal Laser Scanning Microscope (CLSM). It is worth mentioning that, CLSM cannot measure the cuspal deflection precisely five minutes after polymerization because it takes time to scan individual micrographs and combine them to form a complete three-dimensional analysis. This process slightly consumes more time for scanning and thus could affect difference in results obtained between both studies [58]. Moreover, results came also in contradiction with Duarte et al., who also reported no significant difference between incrementally packed and bulk-fill RBCs regarding cuspal deflection.The discrepancy between the present study and their results might also be due to the difference in the utilised methodology, where cuspal deflection was measured ten minutes after polymerization rather than five minutes, or due to the difference in the type of RBC compared [22].

According to literature, microleakage is affected by the anatomy and size of the tooth, cavity C-factor, bonding technique, and light curing technique. In the current study, the previously mentioned factors were standardized to obtain findings that only reflect the impact of the RBC type on microleakage [59].

C-factor refers to the ratio of bonded to unbonded surfaces in prepared cavities. MOD cavities are considered high C-factor cavities, which increases the risk of cuspal deflection and microleakage. As the C-factor rises, cuspal deflection rises, especially in large MOD cavities, due to the stiffness of RBCs that lead to the inward movement of cusps. High C-factor also increases the risk of microleakage because the intense interfacial stress can disrupt the resin-dentin bond. Increasing C-factor restricts the compensation of polymerization shrinkage by monomer relaxation [60].

Moreover, changes in temperature that occur in the oral cavity can aggravate microleakage; thus, the thermocycling technique is utilized in the current study. The thermocycling process accelerates the aging process of restorative material by inducing more permeability of the hybrid layer and increasing restoration porosity. Consequently, the stress that a restoration could exhibit with time can predict in vitro the restoration longevity in vivo. In the current study, thermal aging was conducted through 5000 water baths maintained at 5 °C and 55 °C. This thermocycling protocol was followed to mimic the intraoral thermal fluctuations equivalent to six months of clinical service [61].

Various methodologies were applied in several studies to assess microleakage, encompassing the radioisotope method, acetate peel technique, and dye penetration. These methods are frequently followed by assessment using a stereomicroscope, confocal microscope, or optical coherence tomography [62]. In the current study, the dye penetration technique was utilized for detecting microleakage, as it eliminated the use of any reactive chemicals and radiation. As reported in literature: the procedure is validated, precise and easy to perform with the aid of basic laboratory setup. The 2% methylene blue dye was chosen to be used as it is simple to be utilized, has high water solubility and rapid diffusion in cracks or irregularities in a material. Moreover, each methylene blue dye molecule has a diameter of (0.80 nm) which is less than the dentinal tubule diameter (1–4 μm) and it is reported to have a better dye penetration than butyric acid [63].

In the present study, specimens were sectioned in a single longitudinal direction at the midline, as it is less user sensitive, less time consuming and an easier approach than three-dimensional evaluation [63]. Microleakage was assessed at the gingival margin in this study, as cervical enamel is known to be thinner at this area than at the occlusal margin, thus microleakage is usually more common and evident at the gingival margins of restorations [22].

The results in this study demonstrated that both tested RBC materials could not completely eliminate microleakage. Universal adhesive was used with both RBCs in self-etch mode, which was reported to have higher microleakage scores compared to total etch mode. This could be attributed to the lower acidity of self-etch adhesives compared to 37% phosphoric acid, thus etching enamel and dentin to a lower depth [64]. Incorporated hydrophilic monomers also contribute to resin swelling, water uptake and microleakage [65]. The effect of the adhesion protocol most likely resulted in lower sealing ability and increased microleakage and might have masked the effect of the type of RBC material applied.

Also, the results showed that regardless of thermocycling, Filtek Z250 XT RBC presented insignificantly greater microleakage values than the Grandio^®^SO x-tra RBC. This insignificant increased microleakage value was most likely due to application of multiple successive increments in case of conventional RBCs material. This consequently can form microbubbles between subsequently applied layers, leading to microgaps in the resin-tooth interface and increased dye penetration [59].

These findings are consistent with Cayo-Rojas CF et al., and Mosharafian et al., who found no significant difference between incrementally packed and bulk fill RBCs [37, 59]. The lower marginal microleakage of the tested bulk-fill composite material can be due to its modified composition, higher curing depth, and lower polymerization shrinkage [66]. On the other hand, these results are in contrast to those reported by Somani et al., who found significantly higher microleakage values in bulk-fill RBCs groups in comparison to incrementally packed groups [67]. This could be attributed to Class V cavities utilized in their study, unlike MOD cavities used in the present study.

Regarding the effect of thermocycling on microleakage values, the results of the present study showed that thermocycling recorded significantly higher values in comparison to non-thermocycled subgroups, regardless of the RBC used. These results could be attributed to the fact that thermocycling facilitates hydrolysis and generates recurrent contraction-expansion stresses at the tooth/restoration interface, which lead to gap formation [68]. These findings are consistent with Sonkaya and Yilidrim., Aboelenen et al., Erdilek et al., and Soares et al., who also stated that thermocycling increased the marginal gap between restoration and tooth margins [69–72]. On the other hand, this was contradictory to the results found by Atash et al., who stated that thermocycling had no significant effect on increasing microleakage in specimens. This difference in results may be due to the use of different types of adhesives and bovine rather than human teeth. The resistance to marginal degradation after thermocycling in their study was explained by the different filled adhesives used. The thick adhesive layer obtained with these adhesives allowed the interfaces to maintain adhesion during the critical early stages of polymerization, thereby improving the resistance to dimensional changes [73].

From all the findings mentioned before, the null hypothesis related to microleakage was partially rejected as no significant difference was found in microleakage between incrementally packed and bulk-fill RBCs groups, while thermocycled groups exhibited significantly higher microleakage values compared with the non-thermocycled group.

This study describes the mechanical in-vitro behavior of the tested RBCs; however, future in-vivo validation is required to confirm the findings of this study under clinical conditions. Long-term clinical trials should be carried out to confirm whether reduced cuspal deflection translates to reduced post-operative pain, secondary caries, and tooth fracture. Clinical trials should also evaluate if microleakage translates in-vivo to marginal staining, pulpal inflammation, secondary caries, and restoration failure over long-term follow-ups.

### Limitations and future directions

Multiple limitations in this study could affect the accuracy of results and clinical relevance; although cavities were standardized to the greatest extent possible, minor manual variations in the internal line angles or wall taper can lead to variations in cuspal deflection results. The preparation of MOD cavities in extracted teeth may not exactly simulate the biomechanical behavior of vital maxillary premolars due to the absence of pulpal pressure and natural dentin hydration, which influence the elasticity of remaining tooth structure. Variability in bonding to enamel and dentin may have influenced the findings, as resin-dentin interface is usually more technique sensitive than enamel bonds. This may have masked the effect of tested RBCs on cuspal deflection and microleakage. In addition, the polymerization process continues after the light source is removed, thus delayed shrinkage stresses can still form 24 h following restoration. Restorations in the oral cavity undergo hygroscopic expansion, leading to partial relaxation of the internal stresses with time partially reversing some of the measured deflectionn [29]. Horizontal metroscopes depend on tooth contact probes for measurements, which may apply a minor force on the cusps. The contact probes may shift slightly during measurements, which may affect the accuracy of the measured values. Future protocols should incorporate non-contact measurment methods for a more holistic view of tooth movement. Long-term monitoring to assess water sorption and compensation of polymerization shrinkage with time is advised. Lack of dynamic masticatory loading, which can lead to cuspal fracture and restorative fatigue over time, is also considered a major limitation of the current study. The complex oral environment, including moisture and thermal fluctuations, may induce long-term expansion and contraction, leading to thermal fatigue and microleakage that might not appear in in-vitro studies. Future studies should investigate deeper into newer reinforcement RBCs and their ability to reinforce the cusps as well as maintain marginal integrity.

### Clinical considerations

Bulk-fill RBC showed reduced cuspal deflection in maxillary premolars, which may offer a biomechanical advantage. This suggests that their use could lower the risk of fracture and postoperative pain in clinical practice. While the difference between the two tested materials for microleakage was insignificant, suggesting comparable clinical performance, the significant impact of thermocycling is highly relevant to clinical practice. Clinically, this shows that thermal aging and dynamic oral changes can have a greater deteriorating influence on the resin-dentin interface. These findings emphasize the need for long-term clinical monitoring of restorations.

## Conclusions

Within limitations, this study demonstrated that the low-shrinkage bulk-fill composite reduced cuspal deflection in comparison to the incrementally packed material. Bulk-fill and incrementally packed RBCs both showed comparable vulnerability to microleakage. The thermocycling procedure exerted a deteriorating effect on marginal integrity.

## Data Availability

The datasets used and/or analyzed during the current study are available from the corresponding author upon reasonable request.

## Abbreviations

MOD: Mesio-occluso-distal
10-MDP: 10-Methacryloyloxydecyl dihydrogen phosphate
Bis-GMA: Bisphenol A-glycidyl methacrylate
UDMA: Urethane Dimethacrylate
Bis-EMA: Ethoxylated Bisphenol A Glycol Dimethacrylate
TEGDMA: Triethylene glycol dimethacrylate
TCCDMA: Tricyclodecane Dimethanol Dimethacrylate
ANOVA: Analysis of variance
LED: Light emitting diode

## References

[1] Tosco V, Vitiello F, Furlani M, Gatto ML, Monterubbianesi R, Giuliani A, et al. Microleakage Analysis of Different Bulk-Filling Techniques for Class II Restorations: µ-CT, SEM and EDS Evaluations. J Mater. 2020;14:1–13. 10.3390/ma14010031. 10.3390/ma14010031 PMC7793523 33374708

[2] Ferracane JL. Developing a more complete understanding of stresses produced in dental composites during polymerization. Dent Mater J. 2005;21:36–42. 10.1016/j.dental.2004.10.004. 10.1016/j.dental.2004.10.004 15681000

[3] Duarte RW, Somacal DC, Braga LR, Borges GA, Spohr AM. Cuspal Deflection and Marginal Integrity of Class II Cavities Restored with Bulk-fill Resin Composites. Open Dent J. 2023;17:e187421062309180. 10.2174/0118742106261113230920063110.

[4] Karaman E, Ozgunaltay G. Cuspal Deflection in Premolar Teeth Restored Using Current Composite Resins With and Without Resin-modified Glass Ionomer Liner. Oper Dent. 2013;38:282–9. 10.2341/11-400-L. 23092141 10.2341/11-400-L

[5] Radhika M, Sajjan GS, B N K, Mittal N. Effect of different placement techniques on marginal microleakage of deep class-II cavities restored with two composite resin formulations. J Conserv Dent. 2010;13:9–15. 10.4103/0972-0707.62633. 20582213 10.4103/0972-0707.62633 PMC2883801

[6] Palin WM, Fleming GJP, Nathwani H, Burke FJT, Randall RC. In vitro cuspal deflection and microleakage of maxillary premolars restored with novel low-shrink dental composites. Dent Mater J. 2005;21:324–35. 10.1016/j.dental.2004.05.005. 10.1016/j.dental.2004.05.005 15766579

[7] Urquía-Morales C, Brasca N, Girardi M, Bonnin C, Ríos A, Girardi I, et al. Influence of Surface Sealants on Microleakage in Composite Restorations. Int J Odontostomat. 2017;11:467–73. 10.4067/S0718-381X2017000400467.

[8] Aregawi WA, Fok ASL. Shrinkage stress and cuspal deflection in MOD restorations: analytical solutions and design guidelines. Dent Mater J. 2021;37:783–95. 10.1016/j.dental.2021.02.003. 10.1016/j.dental.2021.02.003 33612308

[9] De Santis R, Lodato V, Gallicchio V, Prisco D, Riccitiello F, Rengo S, et al. Cuspal Deflection and Temperature Rise of MOD Cavities Restored through the Bulk-Fill and Incremental Layering Techniques Using Flowable and Packable Bulk-Fill Composites. Materials. 2020;13:5664. 10.3390/ma13245664. 33322480 10.3390/ma13245664 PMC7763159

[10] Rosatto CMP, Bicalho AA, Veríssimo C, Bragança GF, Rodrigues MP, Tantbirojn D, et al. Mechanical properties, shrinkage stress, cuspal strain and fracture resistance of molars restored with bulk-fill composites and incremental filling technique. J Dent. 2015;43:1519–28. 10.1016/j.jdent.2015.09.007. 26449641 10.1016/j.jdent.2015.09.007

[11] Lu Y, Cai Q, Zhou D. Effect of rapid high-intensity light curing on micro-leakage in conventional and bulk-fill resin composites. Front Mater. 2025;12:1–8. 10.3389/fmats.2025.1578638.

[12] De Mendonça BC, Soto-Montero JR, De Castro EF, Pecorari VGA, Rueggeberg FA, Giannini M. Flexural strength and microhardness of bulk-fill restorative materials. J Esthetic Restor Dent. 2021;33:628–35. 10.1111/jerd.12727. 10.1111/jerd.12727 33675162

[13] Cohen J. Statistical power analysis for the behavioral sciences. 2nd ed. New York: Routledge; 1988. 10.4324/9780203771587.

[14] Faul F, Erdfelder E, Lang A-G, Buchner A. G*Power 3: a flexible statistical power analysis program for the social, behavioral, and biomedical sciences. Behav Res Methods. 2007;39:175–91. 10.3758/bf03193146. 17695343 10.3758/bf03193146

[15] Lakens D. Calculating and reporting effect sizes to facilitate cumulative science: a practical primer for t-tests and ANOVAs. Front Psychol. 2013;4:863. 10.3389/fpsyg.2013.00863. 24324449 10.3389/fpsyg.2013.00863 PMC3840331

[16] Hegde RS, Biradar N, Patil BS, Moogi P, Allappanavar KS, Shetty NK. Evaluation of Marginal Adaptation of Composite Restorations Reinforced with Novel Enamel Inserts (Biofillers) in Class V Cavities. J Contemp Dent Pract. 2021;21:1368–73. 10.5005/jp-journals-10024-2964. 33893260

[17] Dere M, Ozcan M, Gohring TN. Marginal quality and fracture strength of root-canal treated mandibular molars with overlay restorations after thermocycling and mechanical loading. J Adhes Dent. 2010;12:287–94. 20157656 10.3290/j.jad.a17711

[18] Sari C, Bala O, Akgul S, Alp CK. Effect of using different materials and restorative techniques on cuspal deflection and microleakage in endodontically treated teeth. BMC Oral Health. 2025;25:1–13. 10.1186/s12903-025-05693-0. 40000998 10.1186/s12903-025-05693-0 PMC11853680

[19] Mohamed NI, Safy RK, Elezz AFA. Microtensile Bond Strength, Marginal Leakage, and Antibacterial Effect of Bulk Fill Resin Composite with Alkaline Fillers versus Incremental Nanohybrid Composite Resin. Eur J Dent. 2021;15:425–32. 10.1055/s-0040-1721310. 33368067 10.1055/s-0040-1721310 PMC8382449

[20] Mowlood AJ, Ali AH, Mahdee AF. Cusp deflection and fracture strength of root canal filled premolars with two access cavities designs (Conservative vs Traditional). J Clin Exp Dent. 2022;14:e705–11. 10.4317/jced.59460. 36158771 10.4317/jced.59460 PMC9498641

[21] Fathpour K, Salehi A, Samimi P, Fathi A. Evaluating the Color Matching Ability of a Smart Chromatic Technology–Based Composite Resin for Premolar Teeth Restoration. Int J Dent. 2024;1:1–6. 10.1155/2024/5514821. 10.1155/2024/5514821 PMC11452232 39372122

[22] Duarte RW, Somacal DC, Braga LR, Borges GA, Spohr AM. Cuspal Deflection and Marginal Integrity of Class II Cavities Restored with Bulk-fill Resin Composites. TODENTJ. 2023;17:1–8. 10.2174/0118742106261113230920063110.

[23] Yarmohammadi E, Kasraei S, Sadeghi Y. Comparative Assessment of Cuspal Deflection in Premolars Restored with Bulk-Fill and Conventional Composite Resins. Front Dent. 2019;16:407–14. 10.18502/fid.v16i6.3439. 33089241 10.18502/fid.v16i6.3439 PMC7569276

[24] Punia SK, Sharma D, Kumar Y, Agarwal R, Mehta D, Patel P. Comparative Evaluation of the Effect of Fiber Reinforcement on the Cuspal Deflection in Class II Mesio-occluso-distal Cavities Restored with Resin Composite: An In Vitro Study. Dent J Adv Stud. 2025;13:14–20. 10.5005/djas-11014-0072.

[25] Pal PP, Mazumdar D, Bera S, Kar S, Sahay D, Chowdhuri K. Comparative evaluation of incorporation of ferrule in premolars endocrown designs to check any alterations in their fracture resistance: A pilot study. Conserv Dent Endod J. 2024;27:730–6. 10.4103/JCDE.JCDE_277_24. 10.4103/JCDE.JCDE_277_24 PMC11385903 39262600

[26] Gambarini G, Galli M, Morese A, Stefanelli LV, Abduljabbar F, Giovarruscio M, et al. Precision of Dynamic Navigation to Perform Endodontic Ultraconservative Access Cavities: A Preliminary *In Vitro* Analysis. J endod. 2020;46:1286–90. 10.1016/j.joen.2020.05.022. 32553875 10.1016/j.joen.2020.05.022

[27] Karale R, Prathima BJ, Prashanth BR, Shivaranjan NS, Jain N. The effect of bulk-fill composites: Activa and Smart Dentin Replacement on cuspal deflection in endodontically treated teeth with different access cavity designs. Conserv Dent Endod J. 2022;25:375. 10.4103/jcd.jcd_53_22. 10.4103/jcd.jcd_53_22 PMC9520647 36187866

[28] Labib LM, Nabih SM, Baroudi K. Evaluation of cuspal deflection in premolar teeth restored with low shrinkable resin composite (in vitro study). J Int Soc Prev Community Dent. 2015;5:470–5. 10.4103/2231-0762.167725. 26759800 10.4103/2231-0762.167725 PMC4697231

[29] Elmarakby A, Labib L. Influence of Hygroscopic expansion on cuspal deflection of tooth composite restoration (an in vitro study). Egy Dent J. 2018;64:2469–75. 10.21608/edj.2018.76826.

[30] Firouzmandi M, Alavi AA, Jafarpour D, Sadatsharifee S. Fracture Strength and Marginal Adaptation of Conservative and Extended MOD Cavities Restored with Cention N. Int J Dent. 2021;1. 10.1155/2021/5599042. 10.1155/2021/5599042 PMC8279864 34306083

[31] Mohammadzadeh Z, Mohammadipoor HS. In Vitro Fracture Resistance of Permanent Molars with Undermined Walls Restored With Different Materials and Techniques. J Dent Mater Tech. 2021;10:114–20.

[32] Follak A, Miotti L, Lenzi T, Rocha R, Soares F. Self-etch Approach of Universal Adhesives as an Alternative to Minimize Bond Degradation on Sound Dentin vs Caries-affected Dentin over Time. J Adhes Dent. 2021;23:243–52. 10.3290/j.jad.b1367889. 34060304 10.3290/j.jad.b1367889

[33] Jassé FFDA, Alencar CDM, Zaniboni JF, Silva AM, Campos EAD. Assessment of Marginal Adaptation Before and After Thermo-Mechanical Loading and Volumetric Shrinkage: Bulk Fill versus Conventional Composite. Int J Odontostomat. 2020;14:60–6. 10.4067/S0718-381X2020000100060.

[34] Peskersoy C, Sener M, Gurses OB, Erbil E, Turkun M. Evaluation of Proximal Contact Tightness and Contact Area of Posterior Composite Resin Restorations. Apl Sci. 2024;14:1–11. 10.3390/app14188335.

[35] Desai P, Gosh S, Maiti M. Comparision of cuspal deflection & Microleakage in classII restoration with different materials: an invitro study. IJAR. 2024;12:89–97. 10.21474/IJAR01/19431.

[36] Dieckmann P, Baur A, Dalvai V, Wiedemeier DB, Attin T, Taubock TT. Effect of Composite Age on the Repair Bond Strength after Different Mechanical Surface Pretreatments. J Adhes Dent. 2020;22:365–72. 10.3290/j.jad.a44867. 32666062 10.3290/j.jad.a44867

[37] Mosharrafian S, Farahmand N, Poorzandpoush K, Hosseinipour ZS, Kahforushan M. In vitro microleakage at the enamel and dentin margins of class II cavities of primary molars restored with a bulk-fill and a conventional composite. Clin Exp Dent Res. 2023;9:512–7. 10.1002/cre2.729. 36988512 10.1002/cre2.729 PMC10280602

[38] Bilgrami A, Alam MK, Qazi F, ur R, Maqsood A, Basha S, Ahmed N, et al. An In-Vitro Evaluation of Microleakage in Resin-Based Restorative Materials at Different Time Intervals. Polym J. 2022;14:466–80. 10.3390/polym14030466. 10.3390/polym14030466 PMC8838729 35160456

[39] Chowdhary D, Chandra R, Singh S, Tripathi S, Ojha JJ, COMPARATIVE EVALUATION OF MICROLEAKAGE OF DIFFERENT DENTIN REPLACEMENT MATERIALS. Int J Adv Res. 2020;8:593–8. 10.21474/IJAR01/10669.

[40] Karademir SA, Akarsu S. Does preheating affect the microleakage of bulk-fill composite restorations? Int Dent Res. 2024;14:84–92. 10.5577/intdentres.481.

[41] Reeh ES, Messer HH, Douglas WH. Reduction in tooth stiffness as a result of endodontic and restorative procedures. J Endod. 1989;15:512–6. 10.1016/S0099-2399(89)80191-8. 2639947 10.1016/S0099-2399(89)80191-8

[42] González López S, Sanz Chinesta MV, Ceballos García L, de Haro Gasquet F, González Rodríguez MP. Influence of cavity type and size of composite restorations on cuspal flexure. Med Oral Patol Oral Cir Bucal. 2006;11:E536–540. 17072261

[43] Kumari P, Bansal A, Sundaramurthy S, Choudhary K, Niranjan B, Sijeria P. Comparative evaluation of microleakage two types of restorative materials in primary and permanent posterior teeth: an invitro study. IOSR J Dent Med Sci. 2023;22:30–5. 10.9790/0853-2203053035.

[44] Soares CJ. Polymerization Shrinkage Stresses in a Premolar Restored with Different Composite Resins and Different Incremental Techniques. J Adhes Dent. 2013;15:341–50. 10.3290/j.jad.a29012. 23560252 10.3290/j.jad.a29012

[45] Lins RBE, Aristilde S, Osório JH, Cordeiro CMB, Yanikian CRF, Bicalho AA, et al. Biomechanical behaviour of bulk-fill resin composites in class II restorations. J Mech Behav Biomed Mater. 2019;98:255–61. 10.1016/j.jmbbm.2019.06.032. 31280052 10.1016/j.jmbbm.2019.06.032

[46] Mohammed D, al, - Jubori S. Cuspal Deflection in MOD Molars Restored with Bulk-fill Versus Layered Resin Composite Restorations (A Comparative in-vitro Study). Int J Glob Sci Res. 2024;6:1952–71.

[47] Tamse A, Fuss Z, Lustig J, Kaplavi J. An evaluation of endodontically treated vertically fractured teeth. J Endod. 1999;25:506–8. 10.1016/S0099-2399(99)80292-1. 10687518 10.1016/S0099-2399(99)80292-1

[48] Oliveira AA, Ribeiro MLP, Costa PVM, Pereira RD, Versluis A, Veríssimo C. The effect of filling technique on the cuspal strain, polymerization shrinkage stress, enamel crack formation and depth of cure of restored molars. Dent Mater J. 2022;38:1404–18. 10.1016/j.dental.2022.06.033. 10.1016/j.dental.2022.06.033 35787894

[49] Suliman AA, Boyer DB, Lakes RS. Cusp movement in premolars resulting from composite polymerization shrinkage. Dent Mater J. 1993;9:6–10. 10.1016/0109-5641(93)90096-9. 10.1016/0109-5641(93)90096-9 8299873

[50] Ludovichetti FS, Lucchi P, Zambon G, Pezzato L, Bertolini R, Zerman N, et al. Depth of Cure, Hardness, Roughness and Filler Dimension of Bulk-Fill Flowable, Conventional Flowable and High-Strength Universal Injectable Composites: An In Vitro Study. J Nanomater. 2022;12:1951–66. 10.3390/nano12121951. 10.3390/nano12121951 PMC9228197 35745293

[51] Bardocz-Veres Z, Miklós ML, Biró E-K, Kántor ÉA, Kántor J, Dudás C, et al. New Perspectives in Overcoming Bulk-Fill Composite Polymerization Shrinkage: The Impact of Curing Mode and Layering. J Dent. 2024;12:171. 10.3390/dj12060171. 10.3390/dj12060171 PMC11202601 38920872

[52] Haugen HJ, Ma Q, Linskens S, Par M, Mandic VN, Mensikova E, et al. 3D micro-CT and O-PTIR spectroscopy bring new understanding of the influence of filler content in dental resin composites. Dent Mater J. 2024;40:1881–94. 10.1016/j.dental.2024.09.001. 10.1016/j.dental.2024.09.001 39277488

[53] Bucuta S, Ilie N. Light transmittance and micro-mechanical properties of bulk fill vs. conventional resin based composites. Clin Oral Investig. 2014;18:1991–2000. 10.1007/s00784-013-1177-y. 24414570 10.1007/s00784-013-1177-y

[54] Tusi SK, Hamdollahpoor H, Savadroodbari MM, Fathollahi MS. Comparison of polymerization shrinkage of a new bulk-fill flowable composite with other composites: An in vitro study. Clin Exp Dent Res. 2022;8:1605–13. 10.1002/cre2.656. 36062844 10.1002/cre2.656 PMC9760135

[55] Demirel G, Baltacioglu IH, Kolsuz ME, Ocak M, Bilecenoglu B, Orhan K. Volumetric Cuspal Deflection of Premolars Restored With Different Paste-like Bulk-fill Resin Composites Evaluated by Microcomputed Tomography. Oper Dent. 2020;45:143–50. 10.2341/19-019-L. 31283421 10.2341/19-019-L

[56] Politi I, McHugh LEJ, Al-Fodeh RS, Fleming GJP. Modification of the restoration protocol for resin-based composite (RBC) restoratives (conventional and bulk fill) on cuspal movement and microleakage score in molar teeth. Dent Mater. 2018;34:1271–7. 10.1016/j.dental.2018.05.010. 29857989 10.1016/j.dental.2018.05.010

[57] Elsharkasi M, Platt J, Cook N, Yassen G, Matis B. Cuspal Deflection in Premolar Teeth Restored with Bulk-Fill Resin-Based Composite Materials. Oper Dent. 2018;43:E1–9. 10.2341/16-072-L. 29284100 10.2341/16-072-L

[58] Tsujimoto A, Nagura Y, Barkmeier WW, Watanabe H, Johnson WW, Takamizawa T, et al. Simulated cuspal deflection and flexural properties of high viscosity bulk-fill and conventional resin composites. J Mech Behav Biomed Mater. 2018;87:111–8. 10.1016/j.jmbbm.2018.07.013. 30056308 10.1016/j.jmbbm.2018.07.013

[59] Cayo-Rojas CF, Hernández-Caba KK, Aliaga-Mariñas AS, Ladera-Castañeda MI, Cervantes-Ganoza LA. Microleakage in class II restorations of two bulk fill resin composites and a conventional nanohybrid resin composite: an in vitro study at 10,000 thermocycles. BMC Oral Health. 2021;21:619. 10.1186/s12903-021-01942-0. 34861859 10.1186/s12903-021-01942-0 PMC8642901

[60] Ghulman MA. Effect of cavity configuration (C factor) on the marginal adaptation of low-shrinking composite: a comparative ex vivo study. Int J Dent. 2011;2011:159749. 10.1155/2011/159749. 21949664 10.1155/2011/159749 PMC3178442

[61] Morresi AL, D’Amario M, Capogreco M, Gatto R, Marzo G, D’Arcangelo C, et al. Thermal cycling for restorative materials: Does a standardized protocol exist in laboratory testing? A literature review. J Mech Behav Biomed Mater. 2014;29:295–308. 10.1016/j.jmbbm.2013.09.013. 24135128 10.1016/j.jmbbm.2013.09.013

[62] Shetty S, Bhat R, Kini A, Shetty P. Microleakage Evaluation of an Alkasite Restorative Material: An In Vitro Dye Penetration Study. J Contemp Dent Pract. 2019;20:1315–8. 10.5005/jp-journals-10024-2720. 31892684

[63] Abuzaid M, Hamama H. Microleakage of Different Restorative Materials in Class V Restorations: In-Vitro Study. IJSR. 2022;11:720–4. 10.21275/SR22411084020.

[64] Singh S, Bhadauria US, Sharma A, Verma Mathur R. Comparative Evaluation of Microleakage With Total-Etch, Universal (Self-Etch Mode), and Nano Adhesive Systems in Class V Composite Restorations: An In-Vitro Study. Cureus. 2023;15:e46766. 10.7759/cureus.46766. 37954744 10.7759/cureus.46766 PMC10632741

[65] Hurtado A, Fuentes V, Cura M, Tamayo A, Ceballos L. Long-Term In Vitro Adhesive Properties of Two Universal Adhesives to Dentin. Mater (Basel). 2023;16:3458. 10.3390/ma16093458. 10.3390/ma16093458 PMC10179858 37176339

[66] Politi I, McHugh LEJ, Al-Fodeh RS, Fleming GJP. Modification of the restoration protocol for resin-based composite (RBC) restoratives (conventional and bulk fill) on cuspal movement and microleakage score in molar teeth. Dent Mater J. 2018;34:1271–7. 10.1016/j.dental.2018.05.010. 10.1016/j.dental.2018.05.010 29857989

[67] Somani R, Som NK, Jaidka S, Hussain S. Comparative Evaluation of Microleakage in Various Placement Techniques of Composite Restoration: An In Vitro Study. Int J Clin Pediatr Dent. 2020;13:264–8. 10.5005/jp-journals-10005-1764. 32904122 10.5005/jp-journals-10005-1764 PMC7450201

[68] Zanatta RF, Wiegand A, Dullin C, Borges AB, Torres CRG, Rizk M. Comparison of micro-CT and conventional dye penetration for microleakage assessment after different aging conditions. Int J Adhes Adhes. 2019;89:161–7. 10.1016/j.ijadhadh.2019.01.008.

[69] Sonkaya E, Yıldırım ZS. Microleakage of current self-adhesive restorative materials: effect of thermocycling. Int J Dent. 2024;74:121–2. 10.1016/j.identj.2024.07.942.

[70] Aboelenen RH, Mokhtar A, Zaghloul H. Evaluation of marginal fit and microleakage of monolithic zirconia crowns cemented by bio-active and glass ionomer cements: In vitro study. Braz Dent Sci. 2020;23:1824–35. 10.14295/bds.2020.v23i1.1824.

[71] Erdilek D, Dörter C, Koray F, Kunzelmann K-H, Efes BG, Gomec Y. Effect of Thermo-mechanical Load Cycling on Microleakage in Class II Ormocer Restorations. Eur J Dent. 2009;3:200–5. 19756194 PMC2741191

[72] Soares GP, Ambrosano GMB, Lima DANL, Marchi GM, Correr-Sobrinho L, Lovadino JR, et al. Effect of light polymerization time, mode, and thermal and mechanical load cycling on microleakage in resin composite restorations. Lasers Med Sci. 2014;29:545–50. 10.1007/s10103-012-1244-7. 23314786 10.1007/s10103-012-1244-7

[73] Atash R, Shayegan A, Poureslami H, Sharifi H, Shadman N. Effect of Thermocycling on Microleakage of New Adhesive Systems on Primary Teeth: An In-Vitro Study. J Dent Mater Tech. 2013;2:109–13.

